# Black and White Mulberry Extracts: Phytochemical Profile and *In Vitro* Antiatherogenic and Antiplatelet Properties

**DOI:** 10.3390/plants15152394

**Published:** 2026-08-05

**Authors:** Eirini Lantavou, Maria Xenaki, Sofia Bellou, Stavros Beteinakis, Alexios-Leandros Skaltsounis, Alexandros D. Tselepis, Despoina Pantazi, Panagiotis Stathopoulos

**Affiliations:** 1 Atherothrombosis Research Centre, Network of Research Supporting Laboratories (NRSL) & Laboratory of Biochemistry, Department of Chemistry, University of Ioannina, 45110 Ioannina, Greece; elantavou@gmail.com (E.L.); atselep@uoi.gr (A.D.T.); 2 Division of Pharmacognosy & Natural Products Chemistry, Department of Pharmacy, National and Kapodistrian University of Athens, 15771 Athens, Greece; maxenaki@pharm.uoa.gr (M.X.); sbeteinakis@pharm.uoa.gr (S.B.); skaltsounis@pharm.uoa.gr (A.-L.S.); 3 PharmaGnose S.A., Papathanasiou 24, 34100 Chalkida, Greece; 4 Confocal Laser Scanning Microscopy Unit, Network of Research Supporting Laboratories (NRSL), University of Ioannina, 45110 Ioannina, Greece; sofiabellou@uoi.gr

**Keywords:** atherosclerosis, anthocyanins, antiplatelet properties, berries, cardiovascular disease, netosis, oxidation of LDL

## Abstract

Mulberry fruits are rich sources of bioactive phytochemicals with potential cardiovascular-relative bioactivity properties. In the present study, phenolic/alkaloid-enriched extracts from black (*Morus nigra*) and white (*Morus alba*) mulberries were chemically characterized and evaluated for their anti-atherogenic and antiplatelet activities *in vitro*. LC-HRMS analysis revealed distinct phytochemical profiles, with the black mulberry extract (BlackM) being characterized by a high abundance of anthocyanins, predominantly cyanidin- and pelargonidin-based glycosides, whereas the white mulberry extract (WhiteM) was particularly rich in pyrrolidine alkaloids, including morusimic acid isomers. Both extracts also contain flavonoids and phenolic acids, such as rutin, quercetin derivatives, and chlorogenic acid derivatives. BlackM markedly increased the resistance of low-density lipoprotein (LDL) to Cu^2+^-induced oxidation, whereas WhiteM more effectively inhibited the propagation phase of lipid peroxidation and significantly suppressed arachidonic acid-induced platelet aggregation. BlackM induced a modest, albeit non-significant, reduction in neutrophil extracellular trap (NET) formation. To the best of our knowledge, this is among the first studies to comparatively investigate the effects of black and white mulberry extracts on LDL oxidation, platelet aggregation and NETs formation. Overall, these findings support the potential use of mulberry-derived phytochemicals as functional food ingredients and nutraceuticals for the prevention of atherothrombotic cardiovascular disease.

## 1. Introduction

Cardiovascular disease (CVD) is the leading cause of death worldwide, primarily driven by atherosclerosis and subsequent thrombus formation [1]. While dietary habits heavily influence atherosclerotic plaque development [2], public health policies increasingly focus on nutritional education for CVD prevention [3,4]. Strong evidence indicates that a healthy diet—rich in fruits and vegetables, moderate in fish, and low in processed foods—reduces systemic inflammation, oxidative stress, and leukocyte/platelet activation, while enhancing the clearance of circulating microparticles [5]. Consequently, such dietary patterns reduce CVD mortality and delay atherothrombosis progression [6], highlighting the importance of understanding dietary mechanisms to develop novel preventive strategies [7,8].

Fruits generally contribute the most to the human diet in terms of vitamins, dietary fiber and certain minerals [9]. The effects of the multiple and complex actions of polyphenols (anthocyanins, proanthocyanidins, phenolic acids and other phenolic compounds) extend beyond their antioxidant activity with an overall positive impact on health [10,11]. Berries, due to these components, exhibit strong antioxidant effects, having a significant impact on human health [12,13,14]. Specifically, extracts derived from *Morus alba* and *Morus nigra* have been consistently reported to exert potent antioxidant, anti-inflammatory, and cardioprotective effects, which are largely attributed to their high content of anthocyanins, flavonoids, phenolic acids, and other phenolic constituents [15,16,17,18]. Several studies have demonstrated that mulberry-derived polyphenols attenuate lipid peroxidation and enhance low-density lipoprotein (LDL) resistance to oxidative modification, thereby reducing oxidative stress associated with early atherogenic events [19,20]. Furthermore, a recent preclinical study from our group demonstrated that white and black mulberry extracts significantly alleviated myocardial ischemia–reperfusion injury and attenuated the development of atherosclerosis in experimental models, further supporting their cardioprotective and anti-atherogenic potential [21].

Among the phytochemicals identified in mulberry fruits, anthocyanins, flavonoids such as quercetin, rutin, and chlorogenic acid derivatives have been shown to exert vasculo-protective and antiplatelet activities through the modulation of oxidative stress, inflammatory signaling pathways, and platelet activation [22,23,24,25,26]. Notably, *Morus nigra* fruits are particularly rich in anthocyanins, mainly cyanidin- and pelargonidin-based glycosides, which are considered major contributors to their antioxidant and cardioprotective properties [27,28]. Anthocyanins have been shown to effectively scavenge reactive oxygen species, inhibit lipid peroxidation, and protect LDL particles from oxidative modification, thereby attenuating one of the earliest events involved in atherogenesis [29,30]. Moreover, *Morus alba* extracts and isolated constituents such as morusinol significantly inhibit platelet aggregation and arterial thrombus formation in experimental models [31,32].

In addition to their effects on oxidative stress and thrombosis, increasing evidence indicates that several dietary polyphenols, including quercetin and anthocyanins, may suppress neutrophil extracellular trap (NET) formation by modulating ROS-dependent signaling pathways and neutrophil activation [33,34,35,36]. However, direct evidence regarding the effects of mulberry-derived phytochemicals on NETosis remains limited. Therefore, despite the well-documented antioxidant and antiplatelet properties of mulberry extracts, their potential effects on NETosis and their combined anti-atherothrombotic actions remain largely unexplored.

The aim of the present study was to characterize the phytochemical composition of black and white mulberry extracts and to evaluate their potential anti-atherogenic and antiplatelet properties *in vitro*. Specifically, we investigated their effects on copper-induced LDL oxidation, neutrophil extracellular trap (NET) formation, and agonist-induced platelet aggregation in order to clarify the mechanisms underlying their protective effects against atherothrombosis.

## 2. Results

### 2.1. Production and Enrichment of Mulberry Extracts

Freeze-dried fruits of *Morus nigra* (black mulberry, BlackM) and *Morus alba* (white mulberry, WhiteM) were extracted using hydroalcoholic solvent systems optimized for each species. The Ultrasound-assisted extraction process resulted in high recovery yields, reaching 72% for BlackM and 63% for WhiteM (Table 1).

The higher extraction yield observed for *M. nigra* indicates a greater recovery of soluble constituents under the selected extraction conditions. To enrich the extracts in phenolic and other bioactive compounds and thereby remove highly polar matrix components, such as sugars and polysaccharides, the crude extracts were subjected to Amberlite XAD-7 adsorption resin treatment. The overall yield of the enriched fractions corresponded to 6.0 g per 100 g of dried berry fruits for both *M. nigra* and *M. alba*.

The resulting enriched extracts were subsequently used for phytochemical characterization by LC-HRMS and for the evaluation of their biological activities.

### 2.2. Phytochemical Profile of Black and White Mulberry Extracts

**Figure 1:**
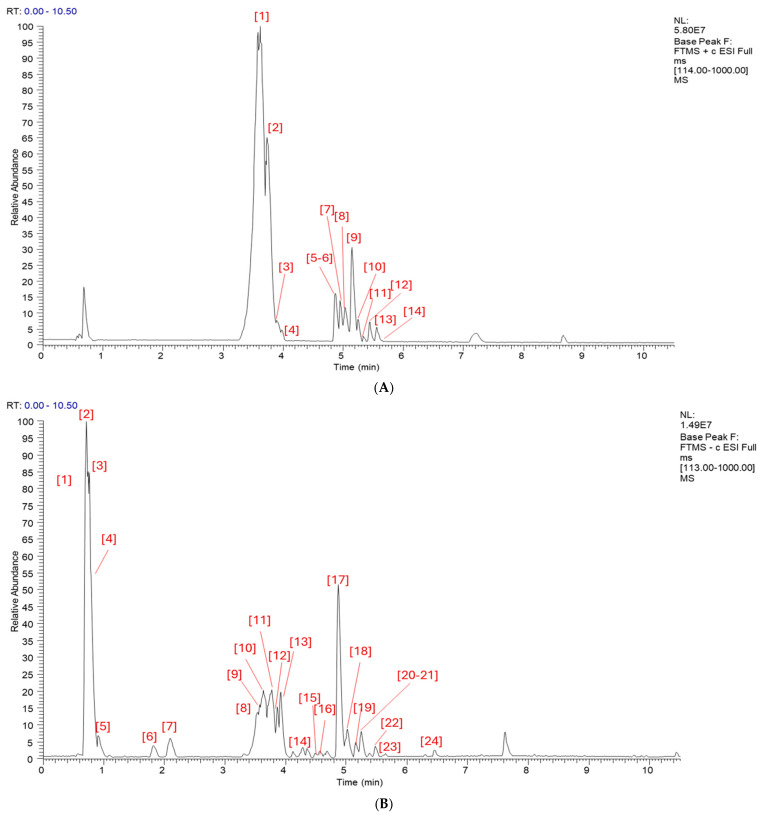
(**A**). UPLC-ESI(+)-HRMS chromatogram of the BlackM extract. Annotated secondary metabolites are highlighted. (**B**). UPLC-ESI(−)-HRMS chromatogram of the BlackM extract. Annotated secondary metabolites are highlighted.

LC-HRMS analysis performed in positive and negative ionization modes enabled the tentative annotation of numerous secondary metabolites in both mulberry extracts. The phytochemical profile of BlackM was characterized by the presence of anthocyanins, flavonoids, phenolic acids, organic acids, and pyrrolidine alkaloids (Figure 1A,B, Table 2). Anthocyanins represented the predominant class of compounds in BlackM, with cyanidin hexoside, cyanidin hexose-deoxyhexose, pelargonidin hexoside, and pelargonidin hexose-deoxyhexose being the major anthocyanins identified. Flavonoids found in the extract included rutin, isoquercitrin, quercetin, kaempferol derivatives, taxifolin, and taxifolin glycosides. In addition, several phenolic acids, including chlorogenic, neochlorogenic, cryptochlorogenic, protocatechuic, vanillic, and *p*-coumaroylquinic acids, were detected. The extract also contained multiple isomers of morusimic acids B, C, and E.

**Figure 2:**
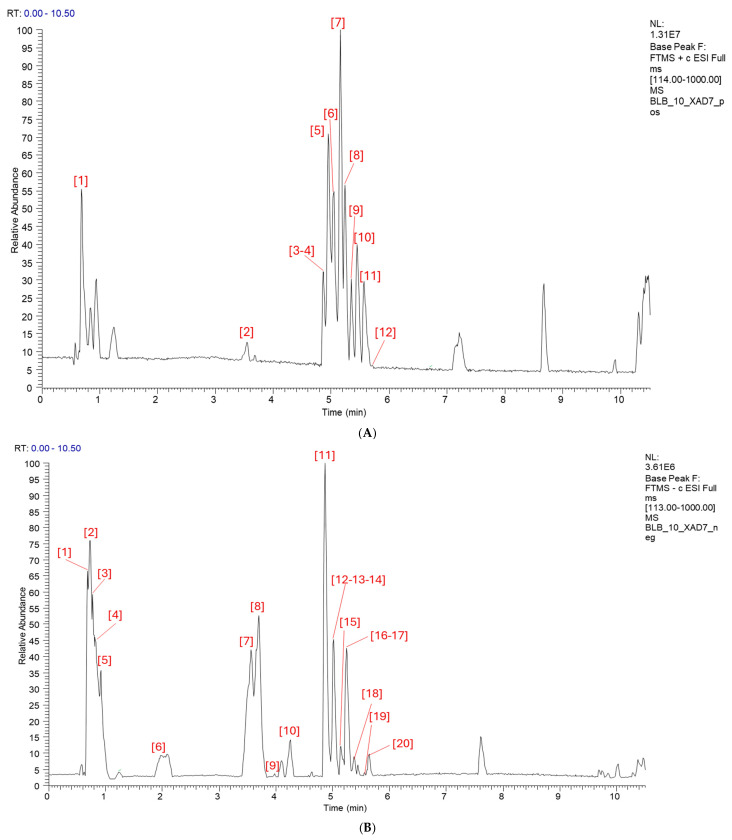
(**A**). UPLC-ESI(+)-HRMS chromatogram of the WhiteM extract. Annotated secondary metabolites are highlighted. (**B**). UPLC-ESI(−)-HRMS chromatogram of the WhiteM extract. Annotated secondary metabolites are highlighted.

WhiteM exhibited a distinct phytochemical profile, characterized predominantly by pyrrolidine alkaloids, together with flavonoids, phenolic acids, and organic acids (Figure 2A,B, Table 3). Among the identified constituents, isomers of morusimic acids B and C represented the most abundant phytochemical components, exhibiting the highest signal intensities in the UPLC-ESI(+)-HRMS chromatogram. The phenolic acid fraction included chlorogenic, neochlorogenic, cryptochlorogenic, *p*-coumaroylquinic, and dicaffeoylquinic acids, whereas quinic, malic, and citric acids were annotated as the major organic acids. Flavonoids detected in the extract comprised rutin, isoquercitrin, quercetin derivatives, kaempferol derivatives, quercetin malonyl hexosides, quercetin acetyl hexoside, and kaempferol 3-*O*-rutinoside. In contrast to BlackM, anthocyanins were not detected in *M. alba*.

Comparison of the two phytochemical profiles revealed the presence of several common metabolites, including rutin, chlorogenic acid derivatives, quercetin glycosides, kaempferol glycosides, and morusimic acid isomers. However, anthocyanins and taxifolin derivatives were exclusively detected in BlackM, whereas dicaffeoylquinic acid derivatives and additional quercetin conjugates were spotted only in WhiteM. Furthermore, semi-quantitative evaluation based on LC-HRMS peak intensities indicated that cyanidin hexoside, cyanidin hexose-deoxyhexose, and pelargonidin glycosides represented the dominant metabolites in BlackM, whereas morusimic acid B/C isomers were among the most abundant constituents detected in WhiteM. These differences in phytochemical composition may account for the differential biological activities observed.

### 2.3. Biological Evaluation of Black and White Mulberry Extracts

#### 2.3.1. Oxidation of LDL

To assess the protective effects of the berry extracts, their ability to increase the resistance of isolated LDL to copper-induced oxidation was evaluated *in vitro*. The kinetic parameters of lipid peroxidation were determined by monitoring the changes in absorbance at 234 nm over time across a range of extract concentrations. Although BlackM extended the lag phase more efficiently (+20 min), it resulted in a 63% reduction in total conjugated dienes (Table 4). In contrast, WhiteM, despite its shorter lag time (+4 min), achieved an 86% inhibition of diene formation. This correlation aligns perfectly with their propagation rates (87% vs. 93% reduction, respectively) (Table 4). It indicates that BlackM acts primarily as a preventative shield extending the lifespan of native LDL, while WhiteM operates as a highly potent chain-breaking scavenger that abruptly terminates lipid peroxidation immediately after initiation (Table 4).

The antioxidant potency and efficacy of the tested berries extracts were also quantified and compared by using IC_50_ and threshold values. BlackM showed significantly lower IC_50_ (23 μg/mL) than WhiteM (92 µg/mL). A lower IC_50_ means greater potency, which means that BlackM requires four times less concentration than WhiteM to achieve the same 50-percent inhibition of LDL oxidation. The relevant threshold values further support this significant difference in potency. Extract BlackM reached its effective antioxidant saturation at a much lower concentration (100 μg/mL) than WhiteM, which required a doubling of the concentration (200 μg/mL) to activate its full antioxidant potential. All of these parameters indicate that BlackM is a highly potent inhibitor that operates optimally at lower, more physiologically relevant doses, while WhiteM requires a higher accumulation to produce comparable overall antioxidant effects.

#### 2.3.2. Aggregation of Platelets

The berry extracts were assessed for their ability to inhibit the aggregation of platelets caused by three agonists: arachidonic acid (AA, 300 μM), adenosine diphosphate (ADP, 10 μM) and TRAP-6 (10 μM). Initially, extracts were examined for potential spontaneous aggregation. None of the tested berry extracts induced platelet aggregation after 15 min incubation at 37 °C. When platelets were stimulated with TRAP-6 (10 μM), none of the extracts showed any appreciable anti-aggregating activity (<20%). When ADP (10 μM) was used as an agonist, WhiteM (400 μg/mL) induced a 38 ± 24% (*n* = 3) inhibition of platelet aggregation, while BlackM had no significant effect (<15%) under the same conditions. Similarly, when platelets were challenged with 300 μΜ of AA, WhiteM at 400 μg/mL showed strong inhibitory activity with a mean of 81 ± 5% (*n* = 3). The strong anti-aggregatory effect of WhiteM was exclusively observed at 400 μg/mL. By contrast, BlackM did not show any significant inhibition (<15%). A possible explanation for this difference is that this specific black mulberry extract may not modulate pathways or membrane receptors that are directly involved in platelet activation.

#### 2.3.3. Netosis

**Figure 3:**
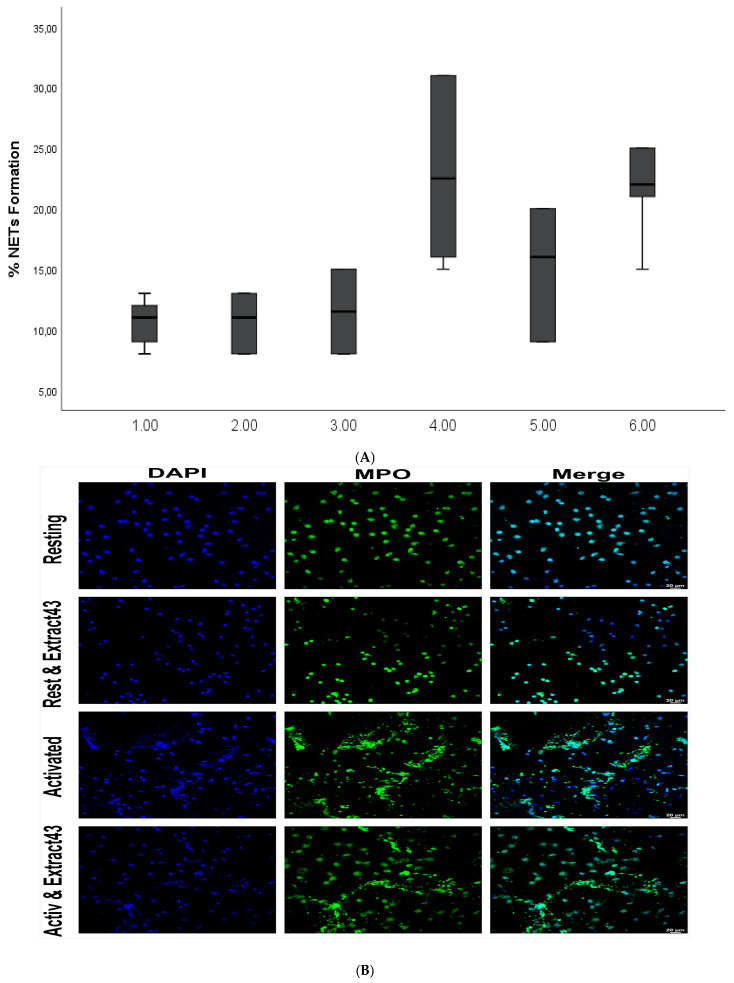
(**A**). Box plots of % NETs formation. 1: Resting; 2: Extract (43 μg/mL); 3: Extract (113 μg/mL); 4: Activated; 5: Activated (43 μg/mL); 6: Activated (113 μg/mL). Data are presented as the Mean ± SD of three different experiments (*p* < 0.05). (**B**). Representative confocal microscopy images from three independent experiments showing neutrophil extracellular trap (NET) formation. DNA was stained with DAPI (blue) and myeloperoxidase (MPO) was detected in green. Neutrophils isolated from healthy volunteers (control) were either untreated or treated with BlackM and examined before and after 3.5 h of PMA stimulation. Images were acquired at 400× magnification. Scale bar, 20 μm.

Given its superior inhibitory activity against LDL oxidation, only the BlackM extract (at concentrations of 43 μg/mL and 100 μg/mL) was further evaluated for its effects on the inhibition of NETs formation *in vitro*. The formation of NETs was confirmed by measuring MPO/DNA levels and also through immunofluorescence imaging (Figure 3A,B).

The BlackM extract in the absence of PMA, did not activate neutrophils to produce NETs (Figure 3A,B). Furthermore, the berry extract at a final concentration of 43 μg/mL reduced NETs formation by 35% (from 23% to 15%, *p* > 0.05) after PMA activation, while at the higher concentration it had no effect on netosis (Figure 3A,B).

**Figure 4:**
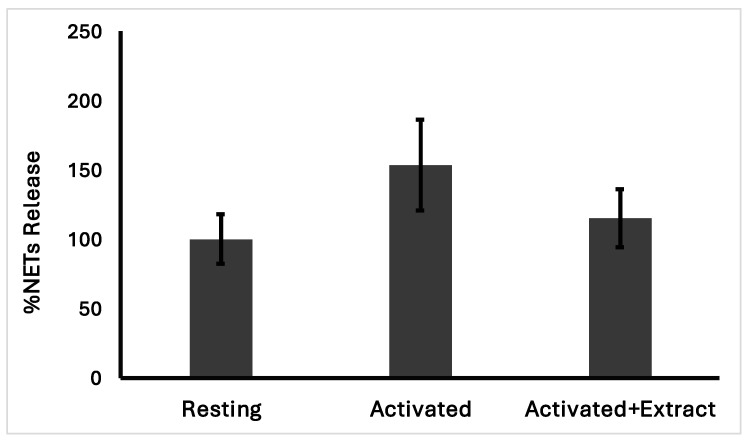
MPO-DNA complex release (%).

Further, extract at concentration 43 μg/mL caused 29 ± 10% inhibition of netosis as determined by MPO-DNA complex levels as measured with our in-house ELISA method. Without the effect of PMA, the extract did not affect netosis (Figure 4).

## 3. Discussion

### 3.1. Production and Phytochemical Characterization of Mulberry Extracts

The extraction protocol employed in the present study resulted in high recovery yields for both mulberry species, with *Morus nigra* and *Morus alba* yielding 72% and 63% crude extracts, respectively. These results suggest that mulberry fruits contain substantial amounts of water-soluble and hydroalcoholic-soluble constituents, including sugars, organic acids, phenolics, and other secondary metabolites [16,17]. The higher extraction yield observed for black mulberry may be attributed to species-specific differences in fruit composition and the greater abundance of polar phytochemicals, particularly anthocyanins, which are characteristic constituents of dark-colored berries [17].

To enhance the concentration of bioactive constituents and reduce the content of undesirable compounds, such as sugars and other non-phenolic components, the crude extracts were further treated using XAD-7 adsorption resin technology. This approach is widely employed for the enrichment of plant extracts owing to its ability to selectively retain phenolic and other aromatic compounds while removing highly polar constituents [37,38]. Following XAD-7 resin treatment, the resulting bioactive metabolite-enriched extracts, referred to as WhiteM and BlackM, corresponded to approximately 6% of the dried fruit weight. The comparable recovery of enriched fractions, despite differences in crude extraction yields, suggests a similar abundance of resin-retained phytochemicals in the two mulberry species.

LC-HRMS analysis revealed marked qualitative differences between the phytochemical profiles of the two mulberry species. BlackM was characterized by the presence of anthocyanins, including cyanidin hexoside, cyanidin hexose-deoxyhexose, pelargonidin hexoside, and pelargonidin hexose-deoxyhexose, compounds that were absent from WhiteM. Anthocyanins are recognized as the principal pigments responsible for the dark coloration of *M. nigra* fruits and have been extensively associated with strong antioxidant, anti-inflammatory, and cardioprotective properties [20,22,27]. The presence of these compounds in BlackM may therefore contribute significantly to the biological activities observed in the present study.

Both extracts were rich in flavonoids and phenolic acids. Among the identified flavonoids, rutin, quercetin derivatives, kaempferol derivatives, and isoquercitrin were detected in both species, while taxifolin and its glycosides were identified only in BlackM. These compounds have been widely reported as potent antioxidants capable of scavenging reactive oxygen species, inhibiting lipid peroxidation, protecting LDL particles from oxidative modification, and modulating inflammatory signaling pathways [23,24]. In addition, chlorogenic acid and related caffeoylquinic acid derivatives, including neochlorogenic and cryptochlorogenic acids, were detected in both extracts, confirming previous reports describing these metabolites as major phenolic constituents of mulberry fruits [16]. Chlorogenic acid exhibits strong radical-scavenging activity and contributes to the preservation of cellular redox balance [25].

An interesting finding was the identification of several pyrrolidine alkaloids, including morusimic acids B, C, and E, primarily in WhiteM. These compounds are considered characteristic metabolites of the genus *Morus* [39] and have attracted increasing interest due to their diverse biological activities. Although the biological activities of morusimic acids remain largely unexplored, pyrrolidine alkaloids from the genus *Morus* have been associated with antioxidant, antidiabetic, and anti-inflammatory properties [40,41]. Therefore, these compounds may contribute, either directly or synergistically with polyphenols, to the overall bioactivity of WhiteM.

The anthocyanin-rich profile of BlackM may partly explain its antioxidant potential. Cyanidin-based anthocyanins have been reported to effectively prevent LDL oxidation by scavenging free radicals and chelating transition metals involved in oxidative reactions [20,22]. Since oxidized LDL is a major contributor to endothelial dysfunction and atherogenesis, the high abundance of anthocyanins together with quercetin and chlorogenic acid derivatives may provide a mechanistic basis for the protective effects observed in the LDL oxidation assay [23,25].

### 3.2. Antiatherogenic and Antiplatelet Activities of Mulberry Extracts

#### 3.2.1. Antioxidant and Antiatherogenic Activity

The distinct phytochemical compositions of BlackM and WhiteM were associated with differential biological activities relevant to atherothrombosis. The LDL oxidation assay, employed in the present study, represents an *ex vivo* model for assessing LDL resistance to oxidative modification rather than a direct measure of cellular antioxidant activity or clinical cardiovascular benefit. Specifically, the anthocyanin-rich BlackM extract exhibited superior potency in protecting LDL against oxidative modification, as evidenced by its lower IC_50_ value and its marked prolongation of the lag phase of LDL oxidation. Anthocyanins, particularly cyanidin-based glycosides, are potent scavengers of reactive oxygen species and effective chelators of transition metals involved in oxidative reactions, thereby reducing lipid peroxidation and preserving the structural integrity of LDL particles [20,25,29]. Moreover, quercetin and chlorogenic acid derivatives identified in BlackM have also been shown to inhibit LDL oxidation and attenuate oxidative stress associated with early atherogenic events [23,25]. Since oxidized LDL promotes endothelial dysfunction, foam-cell formation, and vascular inflammation, the superior antioxidant potency of BlackM may be of particular relevance for the prevention of atherosclerosis [30,42].

Interestingly, despite its lower potency in delaying LDL oxidation, WhiteM more effectively inhibited the propagation phase of lipid peroxidation and markedly reduced total conjugated diene formation. This observation suggests that the antioxidant mechanisms of the two extracts differ. The abundance of pyrrolidine alkaloids, together with chlorogenic acid derivatives and quercetin conjugates, may contribute to potent chain-breaking antioxidant activity by scavenging lipid peroxyl radicals and interrupting the propagation of oxidative reactions. The comparable efficacy of WhiteM in suppressing diene formation further suggests that the biological activities of mulberry extracts are likely mediated by synergistic interactions among their phytochemical constituents rather than by individual compounds alone.

#### 3.2.2. Antiplatelet Activity

The antiplatelet activity observed for WhiteM further highlights the importance of its unique phytochemical composition. WhiteM strongly inhibited arachidonic acid-induced platelet aggregation and moderately suppressed ADP-induced aggregation, whereas BlackM exhibited minimal activity. Previous studies have demonstrated that *Morus alba* extracts and isolated constituents such as morusinol inhibit platelet activation and arterial thrombosis through modulation of intracellular signaling pathways, including ERK and Akt phosphorylation [31,32]. Furthermore, flavonoids identified in WhiteM, particularly quercetin derivatives and rutin, have been shown to inhibit cyclooxygenase-dependent thromboxane generation and attenuate platelet activation [26,43].

The pronounced inhibition of arachidonic acid-induced platelet aggregation observed in the present study therefore suggests modulation of cyclooxygenase-dependent thromboxane A_2_ biosynthesis, a key pathway regulating platelet activation [26,44]. In addition, several phytochemicals identified in the WhiteM extract, including quercetin derivatives, rutin and chlorogenic acid derivatives, have been re-ported to modulate platelet receptor-mediated signaling by attenuating intracellular Ca^2+^ mobilization and downstream activation of ERK, Akt, and phospholipase C pathways [26,45]. Because oxidative stress contributes to platelet activation, the anti-oxidant properties of these phytochemicals may further reduce platelet activation by limiting reactive oxygen species generation and preserving intracellular redox homeostasis [45]. Overall, these mechanisms provide a potential mechanistic explanation for the antiplatelet activity observed in the present study. In addition, plant-derived secondary metabolites are increasingly recognized for their combined antioxidant and anti-inflammatory properties, supporting their potential to modulate multiple signaling pathways involved in platelet activation and vascular inflammation [46].

#### 3.2.3. NETosis and Anti-Inflammatory Activity

Increasing evidence indicates that neutrophil extracellular traps (NETs) contribute significantly to vascular inflammation, endothelial dysfunction, plaque destabilization, and thrombus formation during atherogenesis [33,34,35,36]. Several dietary polyphenols, including anthocyanins, quercetin, and chlorogenic acid, suppress reactive oxygen species production and modulate redox-sensitive signaling pathways involved in NET formation [36]. In the present study, given its superior inhibitory activity against LDL oxidation, only the BlackM extract was further evaluated for its effects on the inhibition of NETs formation *in vitro*. Specifically, BlackM induced a modest, non-significant reduction in PMA-induced NET formation. Although these findings do not support a strong inhibitory effect of BlackM on NETosis, they provide preliminary evidence that mulberry-derived phytochemicals may influence neutrophil activation and justify further mechanistic investigations.

Overall, the present findings indicate that the distinct phytochemical compositions of black and white mulberry extracts are associated with differential antiatherogenic and antiplatelet activities. The anthocyanin-rich BlackM extract exhibited superior potency in protecting LDL against oxidative modification, whereas the alkaloid-rich WhiteM extract exerted pronounced antiplatelet effects. These observations, together with our previous findings demonstrating the cardiovascular-related bioactivity of mulberry extracts in experimental models of myocardial ischemia–reperfusion injury and atherosclerosis [21], further support the potential exploitation of mulberry fruits as sources of functional food ingredients and nutraceuticals for the prevention of atherothrombotic cardiovascular disease.

Although the present findings demonstrate promising *in vitro* biological activities of mulberry-derived phytochemicals, the bioavailability of dietary polyphenols remains an important factor influencing their potential physiological effects. Anthocyanins generally exhibit relatively low intestinal absorption and undergo extensive phase II metabolism, including glucuronidation, sulfation, and methylation, while a substantial proportion reaches the colon, where they are further transformed by the gut microbiota into a variety of low-molecular-weight phenolic metabolites [47,48]. Similarly, chlorogenic acid and flavonoid glycosides are extensively metabolized before entering the systemic circulation [49]. Consequently, the plasma concentrations of intact polyphenols following dietary consumption are typically in the low micromolar or nanomolar range, which may differ considerably from the concentrations used in *in vitro* experiments. Nevertheless, accumulating evidence suggests that both circulating metabolites and microbiota-derived phenolic catabolites retain biological activity and may contribute to the health benefits associated with polyphenol-rich foods. Therefore, further studies evaluating the bioavailability, pharmacokinetics, and biological activity of mulberry-derived phytochemicals in vivo are warranted.

## 4. Materials and Methods

### 4.1. Raw Materials-Berries Collection and Chemicals

White Mulberries (*Morus alba*, Moraceae) and Black Mulberries (*Morus nigra*, Moraceae) were collected from the Attica region of Greece in June 2022. Immediately after collection the berry fruits were transferred to −80 °C, where they remained for one day before being freeze-dried, using a laboratory Freeze-Dryer. All chemicals and high-purity solvents were obtained from Sigma-Aldrich, St. Louis, MO, USA.

### 4.2. Berry Extracts Production

In total, 10.0 g of freeze-dried berry fruits were pulverized, and the resulting powder was extracted with 100 mL of the corresponding solvent mixture. The extraction solvents were selected according to the predominant phytochemical classes present in each mulberry species. Mildly acidic conditions were applied during the extraction of *Morus alba* fruits to facilitate the recovery of pyrrolidine alkaloids, whereas a hydroalcoholic solvent was used for *Morus nigra* fruits to efficiently extract anthocyanins and other phenolic constituents. Specifically, extraction of *Morus alba* fruits was performed using isopropanol/water (70:30, *v*/*v*), whereas *Morus nigra* fruits was extracted with isopropanol/water/acetic acid (60:39.5:0.5, *v*/*v*/*v*). The extraction process was performed in an ultrasonic bath operating at 37 kHz, at 30–40 °C for 30 min. Ultrasonic extraction is well-known and established as a mild, non-thermal, yet highly efficient technique for the recovery of bioactive compounds from plant material [50]. After extraction, the mixture was filtered with a filter paper, and the solid residue was then re-extracted with 100 mL of extraction solvent, following the same procedure. Subsequently the pooled extracts were concentrated using a rotary evaporator (BÜCHI Labortechnik AG, Flawil, Switzerland) under reduced pressure, resulting in the dry berry extracts.

To remove non-active components (e.g., sugars, polysaccharides, polymers) and enrich the extracts with bioactive compounds, XAD-7 adsorption resin technology (ART) was applied. Specifically, 1.0 g of each crude extract was transferred to a conical flask, where 100 mL of water and 10 g of activated XAD-7 resin were added. The mixture was stirred on a magnetic stirrer (IKA-Werke GmbH & Co. KG, Staufen, Germany) for 24 h to adsorb the phenolic compounds onto the surface of the resin. Afterwards, filtration was performed, and the resin was transferred to a conical flask, where 100 mL of methanol was added. The recovery of the XAD-7-bound phenolic compounds from the resin was achieved by placing the conical flask in an ultrasonic bath for 30 min. The methanolic extract was then filtered and concentrated to dryness using a rotary evaporator (BÜCHI Labortechnik AG, Flawil, Switzerland) under reduced pressure.

### 4.3. UPLC-Orbitrap MS/MS Analysis

UPLC-HRMS analysis was performed using a Waters H-Class Acquity UPLC system (Waters, Milford, MA, USA) coupled to a Velos Pro-Orbitrap Elite hybrid mass spectrometer (Thermo Fisher Scientific, Waltham, MA, USA). Chromatographic separation was performed on a Thermo Scientific™ Hypersil GOLD™ C-18 reverse phase column (100 mm × 2.1 mm, 3.0 µm), maintained at 40 °C. The flow rate was set at 400 μL/min and the solvent system was (A) water with 0.1% formic acid and (B) acetonitrile. The gradient elution program started with 5% of B which was held for 1 min. In the next 14 min B reached 100% and was maintained for 2 min. Finally, the system returned to the initial conditions for system equilibration. The injection volume was 10 μL and the autosampler temperature was set at 10 °C.

Mass spectra were obtained in positive and negative ion mode with a heated electrospray ionization (HESI-II) source. The HESI conditions were as follows: capillary and source heater temperature were set at 350 °C; source voltage for positive ion mode at 3.6 kV and for negative at 2.7 kV. Sheath and auxiliary gas were adjusted at 45 and 15 arbitrary units, respectively. HRMS spectra were acquired in full scan mode in the range of *m*/*z* 113-1000, with a resolving power of 60,000 FWHM at *m*/*z* 400. HRMS/MS experiments were obtained in data-dependent acquisition mode with a collision energy of 35.0% (q = 0.25). Specifically, the full-scan acquisition range (*m*/*z* 113-1000) was selected to cover both low-molecular-weight organic and phenolic acids and higher-molecular-weight anthocyanins, flavonoid glycosides, and pyrrolidine alkaloids; HRMS/MS conditions also permitted the detection of diagnostically relevant low-*m*/*z* fragment ions. The raw data were acquired and processed with Thermo Xcalibur Version 2.2. Spectrometric features were used for the identification of constituents, such as accurate mass, proposed elemental composition (EC), observed isotopic patterns and ring double bond equivalents (RDBeq) values. HRMS/MS experiments, in combination with existing in-house and online databases such as the METLIN Metabolomics Database “https://metlin.scripps.edu/ (accessed on 5 June 2026)” and the ChemSpider database “http://www.chemspider.com/ (accessed on 5 June 2026)”, significantly contributed to the annotation of constituents of each extract.

### 4.4. Solubility of Extracts and Storage

The extracts used for the *in vitro* experiments were therefore whole (crude) extracts rather than separate, isolated components. Nevertheless, the removal of sugars during the enrichment process decreases their water solubility. The phytochemical properties of the aqueous extracts before enrichment are also changed by the concentration and redissolution processes, which may also have an impact on their water solubility. The final extracts were therefore only partially soluble in water and diluted in dimethyl sulfoxide (DMSO). Stock solutions were prepared and stored at −20 °C under a nitrogen atmosphere.

### 4.5. Antioxidant Activity

#### 4.5.1. LDL Isolation

LDL (density = 1.019–1.063 g/mL) was isolated from fresh, pooled plasma of normolipidemic volunteers by sequential ultracentrifugation (performed with a Beckman L70 ultracentrifuge (Beckman Coulter, Inc., Brea, CA, USA) at 40,000 rpm and 14 °C using a Type NVT 65 rotor with OptiSeal tubes that contained 0.01% EDTA (*w*/*v*) and also 5 μg/mL penicillin/streptomycin. Thermo Scientific’s Pierce BCA protein assay kit was used to measure the protein content of the separated LDL. LDL was dialyzed extensively against PBS (pH 7.4) at 4 °C to eliminate excess EDTA, as previously described [51]. The increase in absorbance at 234 nm was measured every ten min to determine the kinetics of oxidation for 5 h.

#### 4.5.2. LDL Oxidation

At 37 °C for 5 h, LDL (100 μg protein/mL) was oxidized in the presence of 5 μM CuSO_4_ after being incubated with various concentrations (5–200 μg/mL final concentrations) of berries extracts. An Infinite 200 PRO microELISA reader (Tecan Trading AG, Männedorf, Switzerland) was used to track the oxidation curve at 234 nm [51]. The oxidation curve was used to calculate the threshold and IC_50_ concentration.

### 4.6. Aggregation of Platelets

Platelet Rich Plasma (PRP) was prepared from citrated peripheral venous blood of apparently healthy volunteers. Platelets were adjusted to 250 × 10^6^/mL with homologous Platelet Poor Plasma (PPP) as described previously [52,53,54]. All participants provided written informed consent prior to blood being drawn and the University Hospital of Ioannina’s ethics committee approved the study protocol, which complies with the Declaration of Helsinki.

Berries extracts (40–400 μg/mL) (or their vehicle, DMSO, considered as a control) were added to platelets in 0.5 mL PRP aliquots, and the platelets were pre-incubated for 5 min at 37 °C. Activation of platelets was carried out in a Chronolog Lumi-Aggregometer (Model 700-4DR, Chrono-Log, Havertown, PA, USA) by adding 300 μM AA (Sigma, St. Louis, MO, USA) in EtOH, 10 μM ADP Chrono-log (Havertown, PA, USA) in saline solution, or 10 μM TRAP-6 (Bachem, Bubendorf, Switzerland) in saline solution at 37 °C while continuously stirring at 1200 rpm. The AggroLink software package (ver. 8.0) was used to monitor the aggregation of platelets for 5 min. The inhibitory efficacy of berry extracts was measured and expressed as IC_50_ values in μg/mL, which is the concentration that results in a 50% inhibition of platelet aggregation. In all tests the final concentration of DMSO in PRP was 0.5% (*v*/*v*) [55]. After the blood sample was collected, all aggregation tests were carried out within 4 h.

### 4.7. Netosis

Neutrophils were isolated from heparinized venous blood of apparently healthy volunteers, as described previously [56]. To account for biological variability, neutrophils isolated from three different donors were used in the NETosis experiments. Trypan blue exclusion was employed to assess cell viability prior to stimulation, and only highly viable neutrophil preparations were utilized.

#### 4.7.1. NETs Structures Isolation

A neutrophil suspension was added to a 24-well plate (200,000 neutrophils/well), and a glass coverslip was placed in each well resulting in a final volume of 500 μL of RPMI-1640 culture medium, supplemented with 2% *v*/*v* heat-inactivated human serum. The plate was incubated at 37 °C, in an atmosphere of 5% CO_2_ for 1 h. Neutrophils were incubated with 43 μg/mL and 113 µg/mL of BlackM, or its vehicle (DMSO) for 10 min, and then activated with Phorbol 12-myristate 13-acetate (PMA) (100 nM final concentration) for 3.5 h at 37 °C, 5% CO_2_. All assays included suitable positive and negative controls. To determine whether the compound promotes the formation of Neutrophil Extracellular Traps (NETs) in the absence of PMA stimulation (i.e., spontaneous netosis), the same assay was performed without activating the neutrophils with PMA. In order to evaluate the extract under basal conditions and independently of PMA-induced stimulation, neutrophils were also incubated with the extract in the absence of PMA.

Then, after being fixed with 4% *v*/*v* HCHO, neutrophils were incubated for 1 h in the dark with the primary anti-Myeloperoxidase (MPO) [Santa Cruz Biotechnology, Inc. (Dallas, TX, USA), (266-6K1): sc-52707] and the secondary antibody [goat anti-rabbit IgG (H + L), conjugated with Alexa Fluor^®^ 488, Invitrogen, [(Thermo Fisher Scientific, Waltham, MA, USA), A11034]. After staining the cells with DAPI for 10 min in the dark, the coverslips were mounted on slides and examined by immunofluorescence microscopy using an Olympus BX41 fluorescence microscope equipped with a DP70 camera (Olympus, Tokyo, Japan) at 40× magnification. The number of neutrophils forming NETs was counted in five representative fields, evaluating 200 cells per condition, and the percentage of NETosis was calculated [56].

#### 4.7.2. Isolation of NETs Structures

Primarily, 1.2 × 10^6^ neutrophils were seeded in 6-well culture plates (Corning Incorporated, New York, NY, USA), in RPMI medium. Cell cultures were treated with PMA (100 nM final concentration) for 4 h. Following the treatment period, the medium was removed, and the cells were washed twice with pre-warmed RPMI medium. The NETs structures present in the supernatant were then collected in supernatant after centrifugation at 200× *g* for 5 min [57]. Supernatants were aliquoted and stored at −80 °C in aliquots until the time of use.

#### 4.7.3. Measurement of MPO/DNA Complex

MPO-DNA complexes were measured in NETs structures isolated as described previously [56]. In brief, 5 μg/mL of anti-MPO MAb (Hycult Biotech Inc, Wayne, MI, USA) (dilution, 1:500 with 12.5 mM PBS) was used to coat an F-bottom, with MICROLON 600, high-binding 96-well plates (MICROLON^®^ 600, Greiner Bio-One, Frickenhausen, Germany) overnight at 4 °C. After 3 washes with 12.5 mM PBS, we added 20 μL of isolated NET structures in each well, and the volume was adjusted to 100 μL with incubation buffer containing a peroxidase-labeled anti-DNA MAb (Cell Death ELISAPLUS; Roche Diagnostics, Mannheim, Germany). Plates were incubated for 2 h at room temperature with gentle shaking, followed by 3 washes with 12.5 mM PBS. Subsequently, 100 μL of peroxidase substrate (2,2′-azinobis [3-ethylbenzthiazolinesulfonic acid] [ABTS]) (Cell Death ELISAPLUS; Roche Diagnostics, Mannheim, Germany) was added in each well.

After 20 min of incubation in the dark at room temperature with shaking, the reaction was terminated with 100 μL of ABTS stop solution (Cell Death ELISAPLUS; Roche), and the absorbance was measured at 405 nm using an Infinite 200 PRO microELISA reader (Tecan Trading AG, Männedorf, Switzerland). The release of NETs was expressed as the fold increase in activated cells versus resting neutrophils [58].

### 4.8. Confocal Microscopy

Images were acquired using a Leica TCS-SP5 II scanning confocal microscope, (Leica Microsystems, Wetzlar, Germany) equipped with 405UV/Argon/SS-561/HeNe lasers, using Leica LasX software (Leica Application Suite X (3.10.2.30243)). An APO CS 40.0 × 1.25 OIL UV lens was used throughout the experiment.

### 4.9. Statistical Analysis

Data from at least three independent biological replicates (*n* = 3) were presented as mean ± SD. Statistical analysis was performed with IBM SPSS (version 23; IBM Corporation, Chicago, IL, USA). Student’s *t*-test was used to compare two independent groups of samples. The two-tailed value of *p* < 0.05 was considered statistically significant.

## 5. Conclusions

The present study demonstrated that *Morus nigra* and *Morus alba* (mulberry) fruits are valuable sources of bioactive metabolites with distinct phytochemical profiles and differential *in vitro* biological activities relevant to atherothrombosis. The anthocyanin-rich BlackM extract exhibited superior antioxidant capacity by markedly enhancing the resistance of low-density lipoprotein (LDL) to oxidative modification, whereas the pyrrolidine alkaloid-rich WhiteM extract showed pronounced antiplatelet activity, particularly against arachidonic acid-induced platelet aggregation. Both extracts also contained flavonoids and phenolic acids that may act synergistically and contribute to their observed biological activities. Although BlackM induced only a modest, non-significant reduction in neutrophil extracellular trap (NET) formation, these findings provide preliminary evidence that mulberry-derived phytochemicals may modulate multiple pathways involved in atherothrombosis. To the best of our knowledge, this is among the first studies to comparatively evaluate the effects of black and white mulberry extracts on LDL oxidation, platelet aggregation and NETs formation. Nevertheless, further studies investigating the bioavailability, metabolism, pharmacokinetics, and efficacy of these phytochemicals in animal models and clinical settings are warranted to establish their potential as functional food ingredients or nutraceuticals for supporting cardiovascular health.

## Figures and Tables

**Table 1:** (%) yields of the extracts obtained from *M. alba* and *M. nigra* fruits.

Sample	Extraction Yield (%)	Enriched Extract Yield (g/100 g Dried Fruits)
*Morus nigra*	72	6.0
*Morus alba*	63	6.0

**Table 2:** List of secondary metabolites annotated in BlackM extract, after LC-HRMS analysis in positive and negative ionization mode.

**Peak No**	**Rt (min)**	**Experimental *m*/*z*, [M+H]^+^/[M]^+^**	**RDBeq**	**Molecular** **Formula**	**Error (ppm)**	**HRMS/MS**	**Proposed Compound**
1	3.62	449.1071 [M]^+^	11.5	C_21_H_21_O_11_	−1.643	287.0547	Cyanidin hexoside
2	3.73	595.1640 [M]^+^	12.5	C_27_H_31_O_15_	−2.935	449.1077/287.0549	Cyanidin hexose deoxyhexose
3	3.89	433.1128 [M]^+^	11.5	C_21_H_21_O_10_	−0.285	271.0602	Pelargonidin hexoside
4	3.98	579.1706 [M]^+^	12.5	C_27_H_31_O_14_	−0.400	433.1129/271.0603	Pelargonidin hexose-deoxyhexose
5	4.87	508.3115 [M+H]^+^	2.5	C_24_H_45_NO_10_	−0.242	346.2584	Morusimic acid E
6	4.87	611.1595 [M+H]^+^	12.5	C_27_H_30_O_16_	−1.245	465.1020/303.0495	Rutin (Quercetin-3-O-rutinoside)
7	4.95	492.3158 [M+H]^+^	2.5	C_24_H_45_NO_9_	−1.845	330.2638	Morusimic acid C isomer 1
8	5.03	492.3156 [M+H]^+^	2.5	C_24_H_45_NO_9_	−2.251	330.2635	Morusimic acid C isomer 2
9	5.14	492.3157 [M+H]^+^	2.5	C_24_H_45_NO_9_	−2.048	330.2639	Morusimic acid C isomer 3
10	5.24	551.1026 [M+H]^+^	13.5	C_24_H_22_O_15_	−0.991	303.0498	Quercetin-O- malonylhexoside
11	5.35	330.2638 [M+H]^+^	1.5	C_18_H_35_NO_4_	−0.258	312.2531/268.2635/250.2529	Morusimic acid B isomer 1
12	5.44	330.2635 [M+H]^+^	1.5	C_18_H_35_NO_4_	−1.166	312.2531/268.2635/250.2528	Morusimic acid B isomer 2
13	5.56	330.2638 [M+H]^+^	1.5	C_18_H_35_NO_4_	−0.258	312.2532/268.2635/250.2529	Morusimic acid B isomer 3
14	5.64	535.1082 [M+H]^+^	13.5	C_24_H_22_O_14_	−0.059	287.055	Kaempferol malonyl Hexoside
**Peak No**	**Rt (min)**	**Experimental *m*/*z*, [M-H]−**	**RDBeq**	**Molecular** **Formula**	**Error (ppm)**	**HRMS/MS**	**Proposed Compound**
1	0.65	179.0564	1.5	C_6_H_12_O_6_	0.189	161.0458/134.9878/89.0244	Fructose
2	0.71	195.0507	1.5	C_6_H_12_O_7_	−1.671	177.0406/129.0192/99.0087/87.0087	Gluconic Acid
3	0.74	195.0510	1.5	C_6_H_12_O_7_	−0.133	177.0406/129.0192/99.0087/87.0087	Gluconic Acid
4	0.87	191.0565	2.5	C_7_H_12_O_6_	0.389	-	Quinic acid
5	0.91	191.0199	3.5	C_6_H_8_O_7_	0.174	111.0087	Citric acid
6	1.82	153.0194	5.5	C_7_H_6_O_4_	0.068	109.0292	Dihydroxybenzoic acid (Protocatechuic acid)
7	2.10	353.0873	8.5	C_16_H_18_O_9_	−1.431	191.0561/179.0350/135.0452	Neochlorogenic acid (Caffeoylquinic acid)
8	3.57	353.0881	8.5	C_16_H_18_O_9_	0.835	191.0560/179.0352/135.0451	Chlorogenic acid (Caffeoylquinic acid)
9	3.63	447.0935	12.5	C_21_H_20_O_11_	0.482	327.0513/285.0405	Astragalin (Kaempferol-hexoside)
10	3.70	353.0881	8.5	C_16_H_18_O_9_	0.835	191.0561/179.0349/173.0455/135.0452	Cryptochlorogenic acid (Caffeoylquinic acid)
11	3.77	593.1512	13.5	C_27_H_30_O_15_	0.011	285.0405	Kaempferol hexoside-rhamnoside
12	3.86	611.1608	12.5	C_27_H_32_O_16_	−1.567	475.1457/303.0516/285.0406/241.0509	Taxifolin rutinoside
13	3.91	465.1032	11.5	C_21_H_22_O_12_	−1.396	303.0511/285.0406	Taxifolin hexoside
14	4.23	337.0929	8.5	C_16_H_18_O_8_	0.028	191.0561	3-*p*-Coumaroylquinic acid
15	4.56	303.0511	10.5	C_15_H_12_O_7_	0.244	285.0410/177.0195/125.0246	Taxifolin
16	4.67	167.0351	5.5	C_8_H_8_O_4_	0.706	152.0116/108.0216	Vanillic acid
17	4.87	609.1446	13.5	C_27_H_30_O_16_	−2.475	301.0356	Rutin (Quercetin-3-O- rutinoside)
18	5.02	463.0880	12.5	C_21_H_20_O_12_	−0.43	301.0354	Isoquercitrin (Quercetin-hexoside)
19	5.21	593.1509	13.5	C_27_H_30_O_15_	−0.293	285.0408	Kaempferol 3-O- rutinoside
20	5.25	505.0986	13.5	C_23_H_22_O_13_	−0.324	463.0883/445.0780/301.0355	Quercetin acetyl hexoside isomer 1
21	5.25	549.0884	14.5	C_24_H_22_O_15_	−0.352	-	Quercetin malonyl hexoside
22	5.48	333.0616	10.5	C_16_H_14_O_8_	0.028	165.0191	5,5′-Dehydrodivanillic acid
23	5.64	489.1038	13.5	C_23_H_22_O_12_	−0.101	285.0406	Kaempferol acetyl hexoside
24	6.45	301.0354	11.5	C_15_H_10_O_7_	0.08	178.9985/151.0036	Quercetin

**Table 3:** List of secondary metabolites annotated in WhiteM extract, after LC-HRMS analysis in positive and negative ionization mode.

**Peak No**	**Rt (min)**	**Experimental *m*/*z*, [M+H]^+^**	**RDBeq**	**Molecular** **Formula**	**Error (ppm)**	**HRMS/MS**	**Proposed Compound**
1	0.68	123.0441	5.5	C_7_H_6_O_2_	0.358	95.0491	4-hydroxybenzaldehyde
2	3.55	355.1020	7.5	C_16_H_18_O_9_	−1.010	163.0387	Caffeoylquinic acid
3	4.85	508.3107	2.5	C_24_H_45_NO_10_	−1.816	346.2585/284.2583	Morusimic acid E
4	4.85	611.1594	12.5	C_27_H_30_O_16_	−2.064	465.1022/303.0496	Rutin (Quercetin-3-O-rutinoside)
5	4.95	492.3152	2.5	C_24_H_45_NO_9_	−3.064	330.2635	Morusimic acid C isomer 1
6	5.02	492.3153	2.5	C_24_H_45_NO_9_	−2.861	330.2636	Morusimic acid C isomer 2
7	5.15	492.3152	2.5	C_24_H_45_NO_9_	−3.064	330.2636	Morusimic acid C isomer 3
8	5.24	551.1019	13.5	C_24_H_22_O_15_	−2.261	303.0496	Quercetin malonyl hexoside
9	5.34	330.2631	1.5	C_18_H_35_NO_4_	−2.377	312.2532/268.2635/250.2529	Morusimic acid B isomer 1
10	5.45	330.2631	1.5	C_18_H_35_NO_4_	−2.377	312.2532/268.2635/250.2529	Morusimic acid B isomer 2
11	5.57	330.2633	1.5	C_18_H_35_NO_4_	−1.772	312.2533/268.2636/250.2530	Morusimic acid B isomer 3
12	5.71	535.1071	13.5	C_24_H_22_O_14_	−2.115	287.0549	Kaempferol malonyl hexoside
**Peak No**	**Rt (min)**	**Experimental *m*/*z*, [M-H]−**	**RDBeq**	**Molecular** **Formula**	**Error (ppm)**	**HRMS/MS**	**Proposed Compound**
1	0.69	179.0563	1.5	C_6_H_12_O_6_	1.054	161.0455/89.0243	Fructose
2	0.73	191.0562	2.5	C_7_H_12_O_6_	0.464	173.0456/127.0400/93.0344/85.0293	Quinic acid
3	0.77	215.0329	6.5	C_10_H_8_NaO_4_	1.968	-	Sodium ferulate
4	0.88	133.0144	2.5	C_4_H_6_O_5_	1.154	115.0035/71.0137	Malic acid
5	0.92	191.0198	3.5	C_6_H_8_O_7_	0.388	111.0086	Citric acid
6	1.97	353.0876	8.5	C_16_H_18_O_9_	−0.581	191.0559/173.0456/135.0451	Neochlorogenic acid (Caffeoylquinic acid)
7	3.56	353.0876	8.5	C_16_H_18_O_9_	−0.581	191.0561/135.0453	Chlorogenic acid (Caffeoylquinic acid)
8	3.70	353.0876	8.5	C_16_H_18_O_9_	−0.581	191.0563/173.0456/135.0452	Cryptochlorogenic acid (Caffeoylquinic acid)
9	3.99	711.1404	15.5	C_30_H_32_O_20_	−1.429	667.1511/625.1407/505.0990	Quercetin malonyl dihexoside
10	4.25	337.0931	8.5	C_16_H_18_O_8_	0.621	-	3-*p*-Coumaroylquinic acid
11	4.86	609.1445	13.5	C_27_H_30_O_16_	−2.640	301.0355	Rutin (Quercetin-3-O-rutinoside)
12	5.01	463.0879	12.5	C_21_H_20_O_12_	−0.646	301.0354	Isoquercitrin (Quercetin-hexoside)
13	5.01	625.2137	11.5	C_29_H_38_O_15_	−0.150	-	Quercetin-dihexoside
14	5.01	549.2950	5.5	C_25_H_42_O_13_	−0.481	-	Quercetin malonyl hexoside isomer 1
15	5.21	593.1510	13.5	C_27_H_30_O_15_	−0.326	285.0406	Kaempferol 3-O-rutinoside
16	5.26	505.0985	13.5	C_23_H_22_O_13_	−0.522	463.0882/301.0354	Quercetin acetyl hexoside
17	5.26	549.0882	14.5	C_24_H_22_O_15_	−0.716	-	Quercetin malonyl hexoside isomer 2
18	5.37	515.1191	14.5	C_25_H_24_O_12_	−0.775	353.0879	Dicaffeoylquinic acid isomer 1
19	5.60	515.1190	14.5	C_25_H_24_O_12_	−0.969	353.0879	Dicaffeoylquinic acid isomer 2
20	5.64	489.1037	13.5	C_23_H_22_O_12_	−0.305	285.0404	Kaempferol acetyl hexoside

**Table 4:** Effects of berry extracts on the kinetics of Cu^2+^-induced LDL oxidation.

	Concentration (μΜ)	Lag Time (min)	Reduction in the Oxidation Rate (%)	Reduction in Total Dienes (%)
**LDL**	0	71 ± 6	-	-
***Morus nigra*** **extract**	200	90 ± 2	87	63
***Morus alba*** **extract**	200	75 ± 26	93	86

## Data Availability

The data supporting the findings of this study are available within the article.

## Abbreviations

The following abbreviations are used in this manuscript:

ADP	Adenosine Diphosphate
AA	Arachidonic Acid
DMSO	Dimethyl Sulfoxide
EDTA	Ethylenediaminetetraacetic acid
ESI	Electrospray ionization
HPLC	High Performance Liquid Chromatography
HRMS	High- Resolution Mass Spectrometry
KBr	Potassium bromide
LDL	Low Density Lipoprotein
LTA	Light Transmittance Aggregometry
MPO	Myeloperoxidase
MS	Mass Spectrometry
NETs	Neutrophil Extracellular Traps
PMA	Phorbol Myristate Acetate
PPP	Platelet Poor Plasma
PRP	Platelet Rich Plasma
UHPLC	Ultra High-Performance Liquid Chromatography References
